# Chemotherapy for invasive papillary carcinoma: survival benefit or patient selection?

**DOI:** 10.1093/oncolo/oyag289

**Published:** 2026-08-03

**Authors:** Rashad Ismayilov, Arzu Oguz

**Affiliations:** Department of Medical Oncology, Başkent University Faculty of Medicine, Ankara 06490, Türkiye; Department of Medical Oncology, Başkent University Faculty of Medicine, Ankara 06490, Türkiye

We read with great interest the recent study by Zheng et al., investigating the role of chemotherapy in invasive papillary carcinoma (IPC) of the breast, leveraging the SEER database.^1^ The authors conclude that chemotherapy significantly improves overall survival (OS) in patients with lymph node-negative IPC measuring ≥2.0 cm, thereby advocating for its consideration to prevent undertreatment. While we commend the authors for analyzing a large cohort of this rare subtype, we raise two critical concerns regarding the interpretation of survival data and its clinical applicability.

First, a striking discordance between OS and breast cancer-specific survival (BCSS) outcomes points to significant selection bias. In the propensity score-matched analysis of node-negative patients with tumors ≥2.0 cm, chemotherapy conferred a robust OS benefit (HR: 0.265, *P* < .001). Paradoxically, however, it failed to improve BCSS (HR: 1.233, *P* = .775) (Figure 4C vs. 4D). Had the survival advantage been driven by the cytotoxic eradication of micrometastatic disease, BCSS curves should have diverged in favor of the chemotherapy arm. This discrepancy strongly implies a “healthy candidate bias,” wherein chemotherapy was preferentially administered to fitter patients, while those with significant comorbidities were likely excluded. Crucially, this bias is compounded by the chosen statistical methodology. In a cohort with a median age of 66, death from non-cancer causes represents a major competing event. The reliance on standard Cox regression, which treats non-cancer death as a censored event, risks artificially inflating the cumulative incidence of breast cancer mortality in the older, non-chemotherapy group. A Fine-Gray competing risk regression would provide a more accurate estimation, likely revealing that the perceived “risk” in the non-chemotherapy arm is driven by comorbidities rather than malignancy.^2^

Second, the recommendation to administer chemotherapy based solely on tumor size (≥2.0 cm) risks diverging from contemporary precision medicine. IPC is predominantly an ER-positive, HER2-negative luminal subtype with indolent biology. The landmark TAILORx trial demonstrated that for hormone receptor-positive, HER2-negative, axillary node-negative disease, chemotherapy adds no benefit for the vast majority of patients, even those with larger tumors, provided their genomic recurrence scores are low.^3^ By relying on anatomical staging (tumor size) without genomic context (eg, 21-gene recurrence score), there is a substantial risk of overtreatment. The lack of BCSS benefit in this study further supports the hypothesis that many of these ≥2.0 cm tumors are biologically indolent and adequately managed with endocrine therapy alone.

In conclusion, while retrospective data is vital for rare tumors, the discrepancy between OS and BCSS in this study warrants caution. We suggest that the decision for chemotherapy in node-negative IPC should remain driven by biological risk assessment and genomic profiling, rather than tumor size alone.
